# Optogenetic stimulation of Kiss1^ARC^ terminals in the AVPV induces surge-like luteinizing hormone secretion *via* glutamate release in mice

**DOI:** 10.3389/fendo.2022.1036235

**Published:** 2022-11-08

**Authors:** Xi Shen, Yali Liu, Xiao Feng Li, Hui Long, Li Wang, Qifeng Lyu, Yanping Kuang, Kevin T. O’Byrne

**Affiliations:** ^1^ Department of Assisted Reproduction, Shanghai Ninth People’s Hospital Affiliated to Shanghai Jiaotong University School of Medicine, Shanghai, China; ^2^ Shanghai First Maternity and Infant Hospital, Tongji University School of Medicine, Shanghai, China; ^3^ Department of Women and Children’s Health, Faculty of Life Sciences and Medicine, King’s College London, London, United Kingdom

**Keywords:** LH, optogenetics, projections, ARC, AVPV, kisspeptin

## Abstract

Kisspeptin neurons are mainly located in the arcuate (Kiss1^ARC^, vis-à-vis the GnRH pulse generator) and anteroventral periventricular nucleus (Kiss1^AVPV^, vis-à-vis the GnRH surge generator). Kiss1^ARC^ send fibre projections that connect with Kiss1^AVPV^ somata. However, studies focused on the role of Kiss1^ARC^ neurons in the LH surge are limited, and the role of Kiss1^ARC^ projections to AVPV (Kiss1^ARC→AVPV^) in the preovulatory LH surge is still unknown. To investigate its function, this study used optogenetics to selectively stimulate Kiss1^ARC→AVPV^ and measured changes in circulating LH levels. Kiss1^ARC^ in Kiss-Cre-tdTomato mice were virally infected to express channelrhodopsin-2 proteins, and optical stimulation was applied selectively *via* a fibre optic cannula in the AVPV. Sustained 20 Hz optical stimulation of Kiss1^ARC→AVPV^ from 15:30 to 16:30 h on proestrus effectively induced an immediate increase in LH reaching peak surge-like levels of around 8 ng/ml within 10 min, followed by a gradual decline to baseline over about 40 min. Stimulation at 10 Hz resulted in a non-significant increase in LH levels and 5 Hz stimulation had no effect in proestrous animals. The 20 Hz stimulation induced significantly higher circulating LH levels on proestrus compared with diestrus or estrus, which suggested that the effect of terminal stimulation is modulated by the sex steroid milieu. Additionally, intra-AVPV infusion of glutamate antagonists, AP5+CNQX, completely blocked the increase on LH levels induced by Kiss1^ARC→AVPV^ terminal photostimulation in proestrous animals. These results demonstrate for the first time that optical stimulation of Kiss1^ARC→AVPV^ induces an LH surge-like secretion *via* glutamatergic mechanisms. In conclusion, Kiss1^ARC^ may participate in LH surge generation by glutamate release from terminal projections in the AVPV.

## Introduction

The mid-cycle luteinizing hormone (LH) surge, which is caused by sustained high circulating levels of estradiol, is indispensable for ovulation and reproduction in mammals including humans (1). However, the mechanisms underlying the gonadotropin-releasing hormone (GnRH)/LH surge are not completely understood. GnRH neurons do not express estrogen receptors and therefore unlikely to respond to estrogen directly (2). Kisspeptin (Kiss1) neurons, which are upstream regulators of GnRH neurons, express abundant estrogen receptors and play a key role in the regulation of the hypothalamus-pituitary-gonadal (HPG) axis. The Kiss1 neurons are mainly located in two regions of the rodent brain (3). One population is in the anteroventral periventricular nucleus (AVPV) and the periventricular preoptic nucleus (PeN), and been shown to underlie the pre-ovulatory LH surge, that is plays a ‘GnRH surge generator’ role. While the other population in the arcuate (ARC) co-expresses neurokinin B (NKB) and dynorphin (Dyn), termed KNDy neurons, is considered to be a major component of the GnRH pulse generator (4, 5). The KNDy neurons are also known to be glutamatergic (6–8).

The anatomical link between these two kisspeptin populations has been revealed by several studies using tract-tracing methods and molecular genetics, with Kiss1^ARC^ neurons sending fibre projections to the AVPV region and forming close contacts with the kisspeptin somas in the AVPV (3, 9, 10). These results provide the basis for the close communication between Kiss1^ARC^ and Kiss1 neurons in the AVPV (Kiss1^AVPV^) (3, 9, 10). However, studies focused on the role of Kiss1^ARC^ neurons in the LH surge are limited, with conflicting results (11–13). The magnitude of the LH surge is augmented after ARC neurotoxic ablation (11, 12), which is blocked by dynorphin infusion into the AVPV (11). In contrast, the LH surge magnitude decreases after Kiss1^ARC^ deletion using local micro-injection of antisense kisspeptin cDNA (13).

Various Kiss1-cre transgenic mice models and optogenetic method have been used to explore the role of Kiss1 neurons in the reproductive neuroendocrine system (3, 8, 14, 15). Kiss1^AVPV^ activation can evoke substantial increase in LH comparable to the LH surge (16), while brief Kiss1^ARC^ stimulation results in LH pulses (17, 18) and release different neurotransmitters (6). Most studies have focused on Kiss1^AVPV^ and Kiss1^ARC^ soma stimulation or inhibition *in vitro* and *in vivo*, which would investigate the function of neurons themselves. However, optogenetic stimulation of the neuron terminal projections would be a better way to verify the functionality of specific projections between these two nuclei. Few studies have been conducted on Kiss1^ARC^ terminals activation. Optogenetic stimulation of Kiss1^ARC^ terminals in the preoptic area has been shown to cause hot-flushes (19), but effects on LH surge secretion were not examined.

*In vitro* studies using sagittal brain slices and optogenetic stimulation of fibers from Kiss1^ARC^ neurons demonstrated a glutamatergic input to Kiss1^AVPV^ (6), thus providing clear evidence of a Kiss1^ARC→AVPV^ functional association. Classic studies have shown that glutamate signalling in the AVPV region plays an indispensable role in LH surge generation, confirmed by direct infusion of glutamate agonists or antagonists in this region (20–22). This functionality was further verified by measuring the burst firing rate of Kiss1^AVPV^ neurons *in vitro* with application of glutamate (23). Moreover, glutamatergic input to Kiss1^AVPV^ is augmented by high 17β-estradiol (E_2_) *via* estrogen receptor α (ERα) in the Kiss1^AVPV^, which was confirmed in both KERKO mice (24) and mice with selective deletion of ERα in Kiss1^AVPV^ (25). The glutamatergic afferents to Kiss1^AVPV^ may arise from several loci, one of which may be the Kiss1^ARC^ (26). Most Kiss1^ARC^ neurons (approximately 90%) are glutamatergic (8), and glutamate mRNA expression in Kiss1^ARC^ is increased by E_2_ (27). These findings suggest that Kiss1^ARC^ may participate in the generation of LH surge by facilitating glutamate release from their neuronal projections in the AVPV.

In the present study we used *in vivo* optogenetics technology to investigate the possible function of Kiss1^ARC→AVPV^ projections, by monitoring LH levels after terminal stimulation of Kiss1^ARC→AVPV^ projections. First, different frequencies (5, 10, and 20 Hz) of optic stimulation were used in proestrous mice to determine the most effective activation frequency. Second, Kiss-Cre-tdTomato mice in the diestrous, proestrous and estrous phases of the cycle were used to study the effect of the natural steroid hormone milieu. Finally, to explore the roles of the glutamatergic neurotransmitter released by Kiss1^ARC→AVPV^ projections on LH secretion in proestrus; the combined NMDA and AMPA receptor antagonists (AP5 and CNQX, respectively) were infused unilaterally directly into the AVPV immediately before and during optogenetic stimulation of the Kiss1^ARC→AVPV^ projection terminals.

## Materials and methods

### Animals

Breeding pairs of adult female Kiss-Cre-tdTomato heterozygous transgenic mice (3), which had a special cre activated tdTomato transgene expression in Kiss1 neurons were used. At the same time, wild-type littermates with normal puberty and estrous cyclicality were used as controls. These mice were obtained from the Department of Physiology, Development and Neuroscience, University of Cambridge, UK. Litters from the breeding pairs were genotyped by Polymerase Chain Reaction (PCR), using primers for detecting wild-type: hetF3 (5’-CCG TCA TCC AGC CTA AGT TTC TCA C-3’) and hetR3 (5’-ATA GGT GGC GAC ACA GAG GAG AAG C-3’) and primers for detecting mutant: A526 (5’-GCT TTT ATT GCA CAA GTC TAG AAG CTC-3’) and Asc403 (5’-CAG CCG AAC TGT TCG CCA GGC TCA AGG-3’). Mice were housed individually under a certain temperature (22 ± 2 °C) and a regular light/dark cycle (12:12 h light/dark, lights on 07:00 h). At the same time, the mice were given food (standard maintenance diet; Special Dietary Services, Wittam, UK) and water ad libitum. All animal procedures were approved by a local ethics committee at King’s College London and performed under the regulations of a Home Office License (UK).

### Surgical procedures

Animals were anesthetized using Ketamine (Vetalar, 100 mg/kg, i.p. injection; Pfizer, Sandwich, UK) and xylazine (Rompun, 10 mg/kg, i.p. injection; Bayer, Leverkusen, Germany). All the surgical procedures were conducted using a robot stereotaxic system (Neurostar, Tubingen, Germany). The detailed stereotaxic coordinates used for unilateral injection of channelrhodopsin viral construct in the ARC and implantation of fibre optic or optofluid cannula in the AVPV were obtained from mouse brain atlas of Paxinos and Franklin (28) (ARC: 0.22 mm lateral, 1.30 mm posterior to bregma and at a depth of 6.30 mm; AVPV: 0.20 mm lateral, 0.40 mm anterior to bregma and at a depth of 5.13 mm). Using a 1 μl Hamilton micro-syringe (Esslab, Essex, UK) attached to the stereotaxic frame micro-manipulator, 0.15 μl of the ChR2 virus, AAV9-EF1a-double floxed-hChR2(H134R)-EYFP-WPRE-HGHpA, (≥1x10^13^ GC/ml; Addgene, Massachusetts, USA) was unilaterally injected into the right ARC over 10 min for both Kiss-Cre-tdTomato mice and WT mice. The needle was left in the position for a further 5 min after injection and then withdrawn slowly over 1 min. Then the optic fiber (200 μm, 0.39NA, 1.25mm ceramic ferrule; Thorlabs LTD, Ely, UK) or the optofluid cannula, which contained an optic fiber and fluid injection cannula (Doric Lenses Inc. Quebac, Canada), was implanted into the AVPV and fixed in place using dental cement (Super-Bond Universal Kit, Prestige Dental, UK). Mice were left for 4 weeks preceding the experimental period to allow for effective opsin expression in target regions.

### *In vivo* optogenetic experiments

#### Experiment 1: Different optogenetic stimulation frequency in proestrous mice

Following a one-week recovery period after surgery, the mice were handled daily to acclimatize them to the tail-tip blood sampling procedure for measurement of LH (29). After two weeks following surgery, vaginal cytology was used to check estrous cyclicity (30). Vaginal smears collected at 8:00 - 9:00 h. Only mice in the proestrous stage with regular cyclicity were used. At 14:00 h, the tip of the mouse’s tail was cut using a sterile scalpel, and the fiber optic cannula was then connected through a mating sleeve to a multimode fiber optic rotary joint patch cable (Thorlabs). Animals were allowed to move with freedom. Following a 1 h acclimation period, at 15:00 h, blood samples (5μl) were collected using the tail-tip methods (-30 min) and immediate before optic stimulation (0 min), and then at 10, 20, 30, 40, 50, 60, 75, 90, 120, 150, 180, 210, 240 and 270 min after stimulation onset. After the two baseline blood samples were collected, at 15:30 h (0 min), the optical stimulation started *via* blue light (473 nm wavelength) using a Grass SD9B stimulator controlled DPSS laser (Laserglow Technologies, Toronto, Canada) and was terminated at 16:30 h (60 min). The Kiss-Cre-tdTomato mice (n = 5) received sustained light stimulation (5-ms pulse width, 5mV at tip of patch cable) at 5, 10 or 20 Hz for 60 min. The Kiss-Cre-tdTomato mice receive all 3 optical stimulation protocols on proestrus in a random order with typically one cycle between experiments. The wild type mice (n = 6) received only the highest frequency optical stimulation (20 Hz) as a control.

#### Experiment 2: 20 Hz stimulation in different phases of the estrous cycle

A separate group of Kiss-Cre-tdTomato mice (n = 8) and wild type (n = 5) were used for experiment 2. Estrous cycles stage was evaluated by vaginal smears collected at 8:00 - 9:00 h. Optical stimulation was initiated at 15:30 and ended at 16:30 h as described above for experiment 1. For animals that were in diestrus, proestrus, or estrus, serial blood samples were collected as described in Experiment 1 (For animals in diestrus and estrus, the 240 min blood sample was not collected). In these experiments, Kiss-Cre-tdTomato or wild type (diestrous, proestrous or estrous cycle stage) mice received 20 Hz optical stimulation as described in experiment 1. The experimental order determined randomly across the 3 phases of the estrous cycle and only one stimulation protocol per estrous cycle, with typically 1 cycle between experiments.

#### Experiment 3: 20Hz stimulation at proestrus with intra-AVPV infusion of AP5+CNQX

Neuropharmacological manipulation of glutamatergic signaling was performed in Kiss-Cre-tdTomato animals chronically implanted with optofluid cannulae in the AVPV (n = 7). A combination of NMDA (AP5, Tocris, Abingdon, UK) and AMPA (CNQX, Alpha Aesar, Heysham, UK) receptor antagonist was infused into the AVPV with simultaneous optogenetic stimulation. At 14:00 h on proestrus, an injection cannula connected to extension tubing preloaded with drug solution (AP5 and CNQX dissolved in artificial cerebrospinal fluid; aCSF) or aCSF alone as control, was inserted into the guide cannula of the optofluid assembly. The extension tubing, reaching outside of the cage, was connected to a 5 μl Hamilton syringe mounted in an automated pump (Harvard Apparatus) to allow for remote micro-infusion without disturbing the animals during experimentation. The fiber optic cannula of the optofluid assembly was then connected through a mating sleeve to the multimode fiber optic rotary joint patch cable. Following a 60 min acclimation period, at 15:00 h, serial blood samples were collected as described in Experiment 1. At 15:20 h, 10 min before the onset of optical stimulation (20 Hz for 60 min) a bolus injection of a drug solution (1.2 nmol AP5 and 0.5 nmol CNQX in total 0.4μl aCSF) was given over 10 min, followed by a continuous infusion (2 nmol AP5 and 1 nmol CNQX in total 1μl aCSF) for 60 min (n = 7). The choice of concentrations is according to previous literature (31). Artificial CSF controls, with optical stimulation (20 Hz for 60 min), received the same intra-AVPV fluid regime (n = 7). Treatments were randomized in the experimental design.

### LH measurement

Blood samples were measured using an enzyme-linked immunosorbent assay (ELISA), as previously reported (32). The theoretical limit is 0.117 ng/mL for the LH assay (32). A mouse LH standard (mLH; AFP-5306A, NIDDK-NHPP, USA), coating antibody (RRID: AB_2665514, monoclonal anti-bovine LH beta subunit antiserum, 518B7, University of California, CA, USA), anti-LH antibody (RRID: AB_2665533; National Hormone & Peptide Program, CA, USA) and a secondary antibody (RRID: AB_772206, GE Healthcare, Chicago, Illinois, USA) were used to assess the mice LH concentration. The intra-assay and inter-assay variations were 7.37% and 8.72%, respectively.

### Validation of AAV injection site and fiber optic and optofluid cannula position

Upon completion of experimental procedures, the mice were sacrificed with a lethal dose of ketamine and transcardially perfused by heparinized saline for 5 min, followed by 10 min of ice-cold 4% paraformaldehyde (PFA) in phosphate buffer using a pump. These brains were collected quickly and post-fixed sequentially at 4 °C in 15% sucrose with PFA and 30% sucrose with phosphate buffer until they sank. Brains were then snap frozen on dry ice and stored in -80°C until processing. Brains were coronally sectioned (30μm) between +0.5 mm and -2.7 mm from the bregma and sections were mounted on microscope slides, air-dried and covered slips using Prolonged Antifade mounting medium (Molecular Probes, Inc. OR, USA). The position of injection and fiber was validated by an Olympus FV3000 confocal microscope. The co-localization of red specific fluorescence (Kiss1^ARC^ with tdTomato) and EYFP fluorescence showed that successful virus with ChR2 expression in Kiss1^ARC^. Only the results of mice with correctly positioned fibre optic or optofluid cannulae in the AVPV and EYFP fluorescence in the ARC were included in the analysis.

### Statistical analysis

Statistical comparisons of the mean LH values from 15:00 to 20:00 h were performed using one-way ANOVA followed by *post hoc* Dunnett’s multiple comparison test to compare the basal LH levels (15:00 h: -30 min and 15:30 h: 0 min) with those during and after optogenetic stimulation. The LH level comparison between groups used two-way repeated-measure ANOVA followed by the Holm-Sidak *post hoc* method. The calculation of area under the curve (AUC) for the stimulation period was from 15:30 to 16:30 h, and for the spontaneous LH surge in WT mice from 17:30 to 20:00 h. The AUC was compared between groups used the Mann-Whitney test and two-way repeated-measure ANOVA. Data are presented as the means ± SEM, and *P*<0.05 was considered statistically significant.

## Results

### Validation of AAV injection site and fiber optic and optofluid cannula position

**Figure 1:**
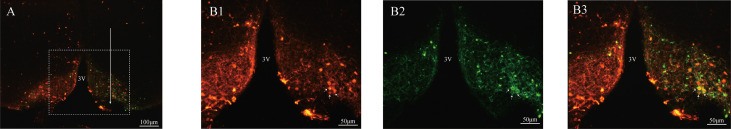
Expression ChR2-EYFP in ARC kisspeptin neurons of Kiss-Cre-tdTomato mice. The brain sections were obtained from Kiss-CRE;tdTomato mice to verify the co-expression of ChR2-EYFP with Kisspeptin neurons. The injection tract in the ARC is shown by the white arrow in **(A)**. **(B1–B3)** Represent the magnified area of the region shown by the white box in **A**, and illustrate the Kiss1^ARC^ neurons (red), ChR2-EYFP expressing neurons (green) and ChR2-EYFP co-localized with Kiss1^ARC^ neurons (yellow) respectively.

**Figure 2:**
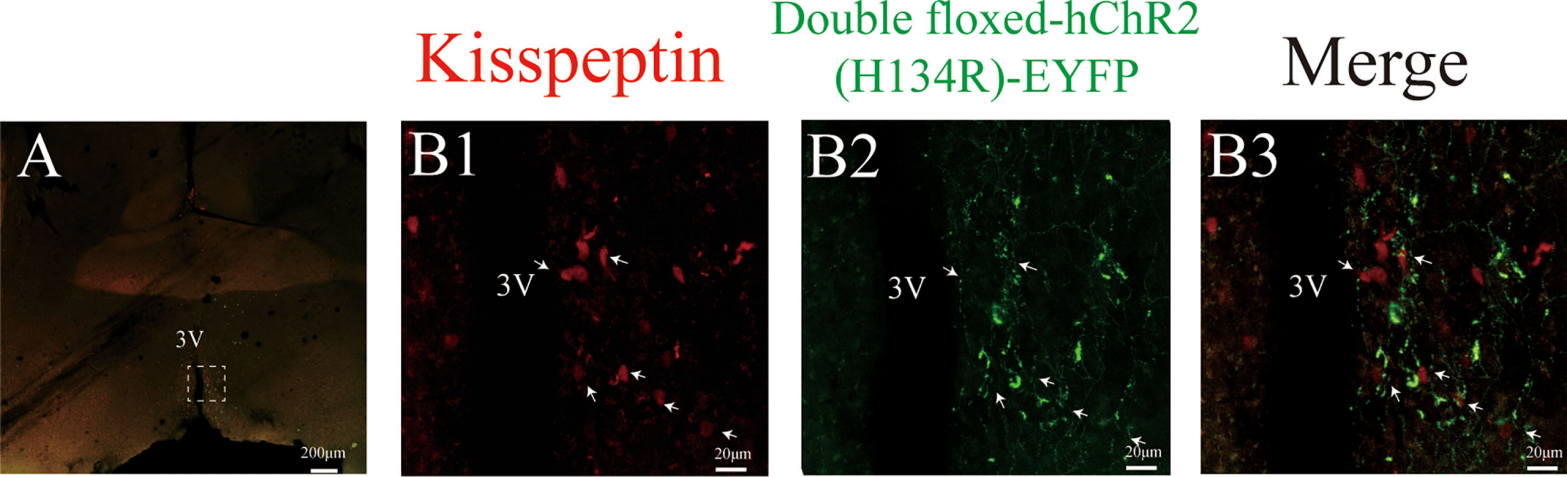
Expression ChR2-EYFP of Kiss1^ARC^ projection terminals in AVPV (Kiss1^ARC→AVPV^) of Kiss-Cre-tdTomato mice. **(A)** Shows the 4X magnification of AVPV area in Kiss1-tdTomato mice. **(B1-B3)** represents the magnified area of the region shown by the white box in A, illustrating the Kiss1^AVPV^ neurons (red), Kiss1^ARC^ terminals in the AVPV region with ChR2-EYFP expression and co-localization in the AVPV area (40X magnification) respectively. The white arrow represents the somata of tdTomato-expressing Kiss1^AVPV^ neurons alongside the projections of ChR2-expressing Kiss1^ARC^ neurons **(B3)**.

The CRE-dependent AAV was injected into the ARC to infect Kiss1^ARC^ neurons with fluorescent tagged ChR2, which could be visualised under a microscope. The Kiss1 neurons in the ARC region carried a tdTomato fluorescence, which is observed bilaterally in the ARC (**Figure 1A**). **Figure 1A** showed the virus with EYFP fluorescence was only observed unilaterally on the injection side and the injection trace is represented *via* the white arrow. The unilateral Kiss1^ARC^ neurons co-expressed AAV-ChR2-virus successfully, which were observed in lower (**Figure 1A**) and higher magnification (**Figures 1B1-B3**). The AAV-ChR2-virus expressed in Kiss1^ARC^ neurons was also found in projection fibres (**Figure 2A**). The terminal projection fibers with EYFP fluorescent were observed in the ipsilateral AVPV region (**Figure 2B2**). Naturally, the Kiss1^AVPV^ neurons are labelled with tdTomato fluorescent (**Figure 2B1**). The EYFP staining projecting fibres from the Kiss1^ARC^ were shown to from close contacts with Kiss1^AVPV^ neurons (**Figure 2B3**). There were sporadic Kiss1^AVPV^ neurons (<5%) that showed EYFP fluorescence, possibly because of a high concentration of virus injection.

Mice with correctly positioned fiber optic or optofluid cannulae in the AVPV and EYFP fluorescence in the ARC included 4 of the 5 Kiss-Cre-tdTomato and 5 of the 6 wild type mice receiving optic stimulation in experiment 1; 7 of the 8 Kiss-Cre-tdTomato and 4 of the 5 wild type mice receiving optic stimulation in experiment 2; 6 of the 7 Kiss-Cre-tdTomato mice receiving AP5+CNQX or aCSF intra-AVPV infusion during optic stimulation in experiment 3. All mice used in the experiment were checked with regular cyclicity (at least three complete estrus cycles).

### Effect of different optogenetic stimulation frequency on LH secretion in proestrous mice

**Figure 3:**
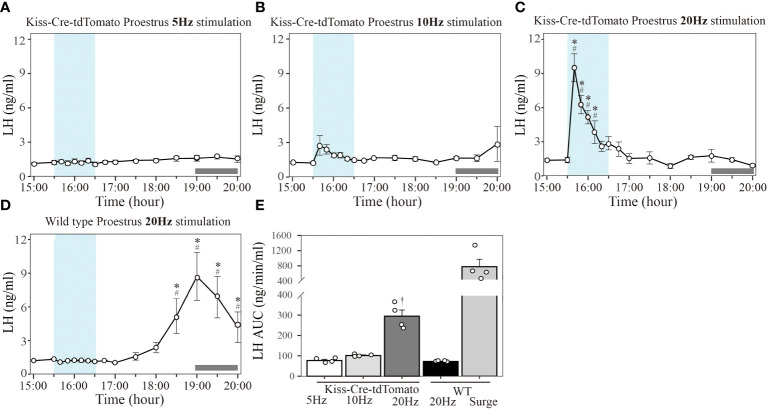
Effect of different frequency of optogenetic stimulation of Kiss1^ARC^ neuron terminals in the AVPV on LH secretion in proestrous mice. Circulating levels of LH (Mean ± SEM) in response to: **(A)** 5 Hz optical stimulation in proestrous Kiss-Cre-tdTomato mice (n = 4), **(B)** 10 Hz stimulation in proestrous Kiss-Cre-tdTomato mice (n = 4), **(C)** 20 Hz stimulation in proestrous Kiss-Cre-tdTomato mice (n = 4) and **(D)** 20 Hz stimulation in proestrous wild type (WT) mice (4/5 mice showed a spontaneous LH surge). **(E)** The AUC for LH secretion during a 1-h optic stimulation (15:30 – 16:30 h) at 5, 10 and 20 Hz in Kiss-cre mice, and 20 Hz stimulation in WT mice. Individual data points were shown as black circles. AUC for the spontaneous LH surge (17:30 – 20:00 h) in WT mice is also indicated in **(E)**. Grey bar on abscissa represents the light-off. *P < 0.05 represents significant difference relative to baseline LH levels at 15:00 h (-30 min). ^#^P < 0.05 represents significant difference of 20 Hz stimulation group relative to 5 Hz and 10 Hz at the same time point. ^†^P < 0.05 relative to other LH AUC treatment groups.

In Kiss-Cre-tdTomato mice sustained 20 Hz stimulation of the Kiss1^ARC^ terminals in the AVPV from 15:30 to 16:30 h on proestrus effectively induced an immediate increase in LH reaching peak surge-like levels of about 8 ng/ml within 10 min, followed by a gradual decline to baseline over a period of about 40 min (**Figure 3C**; n = 4). Stimulation at 10 Hz resulted in a non-significant increase in LH levels (**Figure 3B**; n = 4) and 5 Hz stimulation had no effect in proestrus animals (**Figure 3A**; n = 4). For quantification of the LH response to optic stimulation, AUC in the 1-h stimulation period (15:30-16:30 h) was compared. AUC for 20 Hz stimulation (294.67 ± 30.47 ng/min/ml) was significantly greater that for 10 Hz (101.15 ± 3.04 ng/min/ml) and 5 Hz (77.01 ± 5.67 ng/min/ml), with no difference between the two lower frequency groups (**Figure 3E**). Unexpectedly, the optogenetically stimulated proestrous animals did not show a spontaneous LH surge (**Figure 3C**).

Wild type mice did not show any LH response to 20 Hz optical stimulation in the proestrus stage (**Figure 3D**), but a spontaneous LH surge was observed at the expected time in 4 of the 5 mice (onset at approximately 17:30 h and reaching peak LH level (8.70 ± 2.13 ng/ml) at 19:00 h (**Figure 3D**, n=4). The AUC for the spontaneous LH surges (780.06 ± 194.25 ng/min/ml calculated over the period 17:30-20:00 h) is shown in **Figure 3E**.

The mice excluded from the analysis in experiment 1 (one Kiss-Cre-tdTomato and one WT mouse) was due to an incorrect fiberoptic cannula placement. These mice showed spontaneous LH surges on proestrus (peaking at about 1900h), but no change in LH secretion during optical stimulation.

### Effect of optic stimulation at 20 Hz in different stage of the estrous cycle

**Figure 4:**
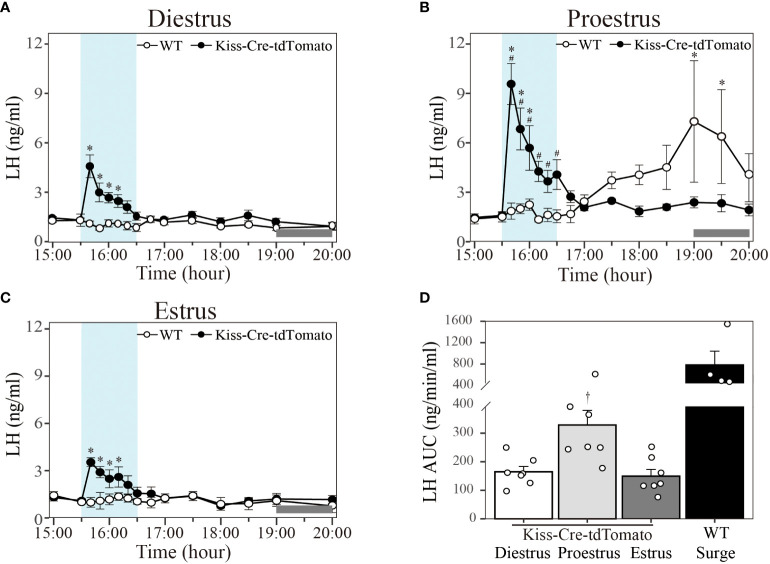
Effect of 20 Hz optical stimulation of Kiss1^ARC^ neuron terminals in the AVPV on LH secretion in different stage of the estrous cycle. Circulating levels of LH (Mean ± SEM) in response to: **(A)** 1-h optic stimulation (15:30 – 16:30 h) in diestrous Kiss-Cre-tdTomato (black circle) and wild type (white circle) mice, **(B)** 1-h optic stimulation (15:30 – 16:30 h) in proestrous Kiss-Cre-tdTomato (black circle) and wild type (white circle) mice and **(C)** 1-h optic stimulation (15:30 – 16:30 h) in estrous Kiss-Cre-tdTomato (black circle) and wild type (white circle) mice. **(D)** The AUC for LH secretion during a 1-h optic stimulation (15:30 – 16:30 h) at 20 Hz in diestrous, proestrous and estrous Kiss-Cre-tdTomato mice. Individual data points were shown as black circles. AUC for the spontaneous LH surge (17:30 – 20:00 h) in WT mice is also indicated in **(D)**. Grey bar on abscissa represents the light-off. *P < 0.05 relative to baseline LH levels at 15:00 h (-30 min). ^#^P < 0.05 represents LH levels in proestrous group relative to mean LH levels in diestrous and estrous at the same time point. ^†^P < 0.05 relative to LH AUC for diestrus and estrus. Kiss-Cre-tdTomato, n = 7 per treatment group. Wildtype, n = 4 per treatment group.

In Kiss-Cre-tdTomato mice sustained 20 Hz stimulation of the Kiss1^ARC^ terminals in the AVPV from 15:30 to 16:30 h evoked an immediate increase in the LH level peaking at 10 min in diestrus (**Figure 4A**; peak LH levels: 4.61 ± 0.69 ng/ml; n = 7), proestrus (**Figure 4B**; peak LH levels: 9.57 ± 1.25 ng/ml; n = 7) and estrus (**Figure 4C**; peak LH levels: 3.54 ± 0.28 ng/ml; n = 7) and lasting for approximately 30-50 min. To compare the LH response to optical stimulation in the three stages of the estrous cycle, AUC in the 1-h stimulation period (15:30-16:30 h) was quantified. AUC for 20 Hz stimulation in proestrus (328.63 ± 51.34 ng/min/ml) was significantly greater that in diestrus (164.74 ± 18.93 ng/min/ml) and estrus (149.83 ± 23.51 ng/min/ml), with no difference between the diestrus and estrus groups (**Figure 4D**). As observed above, these optogenetically stimulated proestrous animals also failed to show a spontaneous LH surge (**Figure 4B**). Wild type mice showed no LH response to 20 Hz optic stimulation in all three stages of the estrous cycle (**Figures 4A–C**; n = 4 per group), but a spontaneous LH surge was observed at approximately 17:30 h (about 1.5 h before lights off) in the proestrus animals, peaking at 19:00 h (7.30 ± 3.70 ng/ml) (**Figure 4B**; n = 4). The AUC for the spontaneous LH surges (781.63 ± 259.66 ng/min/ml calculated over the period 17:30-20:00 h) is shown in **Figure 4D**. The mice excluded from the analysis in this experiment (one Kiss-Cre-tdTomato and one WT mouse) was due to incorrect fiberoptic cannula placement. These mice showed spontaneous LH surges on proestrus (peaking at about 1900h), but no change in LH secretion during optical stimulation.

### Intra-AVPV infusion of the glutamate antagonists blocks optic stimulated LH release in proestrous mice

**Figure 5:**
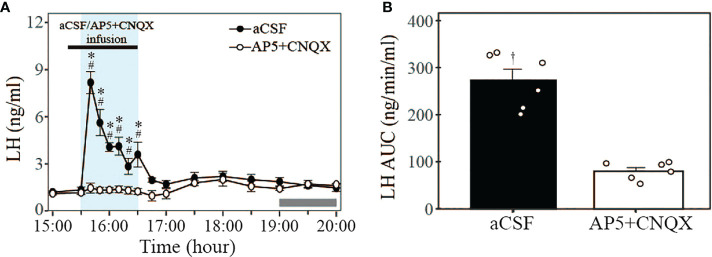
Effect of glutamate antagonism on LH secretion induced by 20 Hz optical stimulation of Kiss1^ARC^ neuron terminals in the AVPV in proestrous Kiss-Cre-tdTomato mice. **(A)** Circulating levels of LH (Mean ± SEM) in response to 1-h 20 Hz optical stimulation (15:30 – 16:30 h) in the presence of intra-AVPV administration of glutamate antagonists (AP5+CNQX) (white circle) or artificial cerebrospinal fluid (aCSF) as control (black circle). The combined NMDA and AMPA receptor antagonists were infused from 15:20 to 16:30 h (black rectangle, bolus over 10 min followed by continuous infusion over 60 min: AP5, 1.2 + 2.0 nmol; CNQX, 0.5 + 1.0 nmol, respectively). **(B)** The AUC for LH secretion during a 1-h optic stimulation (15:30 – 16:30 h) at 20 Hz in the presence of intra-AVPV administration of AP5+CNQX or aCSF in proestrous Kiss-Cre-tdTomato mice. Individual data points were shown as black circles. Grey bar on abscissa represents the light-off. *P < 0.05 relative to baseline LH levels at 15:00 h (-30 min). ^#^P<0.05 represents significant differences of LH levels in control group relative to antagonist group. ^†^P < 0.05 LH AUC for AP5+CNQX vs aCSF. n = 6 per treatment group.

To determine a possible role for glutamatergic neurotransmitter released by Kiss1^ARC→AVPV^ projections on LH secretion at proestrus, the combined glutamate antagonists AP5 and CNQX were infused into the AVPV using the optofluid cannula 10 min before and during the 1-h 20 Hz optogenetic stimulation (15:30-16:30 h) of the Kiss1^ARC→AVPV^ projection terminals *via* a microinfusion pump. Glutamate antagonism completely blocked the optogenetically stimulated LH secretion evident in the aCSF infused controls (peak LH levels: 8.18 ± 0.70 ng/ml) (**Figure 5A**; n = 6 per group). The AUC for LH levels during stimulation in the AP5+CNQX and aCSF infusion groups are shown in **Figure 5B** (AP5+CNQX: 79.77 ± 7.75 vs. aCSF: 272.87 ± 23.58 ng/min/ml). These results show that the glutamate antagonism effectively blocked the LH surge-like secretion induced by optical stimulation. Interestingly both treatment groups failed to show a spontaneous LH surge (**Figure 5A**). The one mouse excluded from the analysis in these experiments was due to a misplaced AAV injection and no ChR2-EYFP expression in the Kiss1^ARC^ neurons, although the optofluid cannula was correctly positioned in the AVPV. This animal showed no LH response to optical stimulation in the presence of aCSF or antagonist, but did display spontaneous LH surges peaking at 1900h.

## Discussion

The present study explored the potential functional link of Kiss1^ARC^ neuron projections to AVPV Kiss1 neurons (Kiss1^ARC→AVPV^) in controlling of the pre-ovulatory LH surge and demonstrated that terminal stimulation of Kiss1^ARC→AVPV^ resulted in an LH surge-like secretion. This is the first study to assess the function of Kiss1^ARC→AVPV^ projections *via* terminal stimulation *in vivo* using an optogenetics approach combined with neuropharmacology. The increased levels of circulating LH induced by 20 Hz optical stimulation in proestrus were significantly greater than those observed in diestrous and estrous mice, suggesting that the neuropeptides/neurotransmitters released by Kiss1^ARC→AVPV^ terminal stimulation were regulated by the sex steroid milieu. Moreover, we demonstrated that administration of the glutamate antagonist combination, AP5 and CNQX, into the AVPV completely blocked the surge-like release of LH induced by 20 Hz Kiss1^ARC→AVPV^ terminals in proestrous mice. The combined NMDA and AMPA receptor antagonists were used since the properties of ionotropic glutamate receptors dictate inhibition of individual receptor type would be less complete (7, 33). These data suggest that the Kiss1^ARC^ terminals in the AVPV region may excite Kiss1^AVPV^ neurons *via* glutamate to regulate the pre-ovulatory LH surge and complement previous *in vitro* studies showing a direct robust excitatory glutamatergic input to Kiss1^AVPV^ neurons from Kiss1^ARC^ (6).

Previous studies on the role of Kiss1^ARC^ in control of the LH surge included anatomical and functional perspectives. Kiss1^ARC^ do not directly interact with the GnRH neurons in the preoptic area (6, 34). It has been demonstrated that Kiss1^ARC^ neurons send fibres to the AVPV region ipsilaterally (3, 9, 10, 34) and form close contacts with Kiss1^AVPV^ (10), which is consistent with our results. Studies examining the potential functional role of Kiss1^ARC^ in the pre-ovulatory LH surge are limited with conflicting results. Previous *in vitro* studies showing that *c-fos* expression in Kiss1^ARC^ remained unchanged and Kiss1^ARC^ mRNA decreased during proestrus are difficult to reconcile with a significant role for Kiss1^ARC^ in generation of the LH surge (35). However, subsequent research demonstrated that *Slc17a6* mRNA (encoding vesicular glutamate transporter 2, necessary for glutamate release) in Kiss1^ARC^ is increased by estrogen (27), which suggests that Kiss1^ARC^ neurons could release more excitatory glutamatergic neurotransmitters in a high estrogen environment. Previous functional *in vivo* studies have also demonstrated a potential role for Kiss1^ARC^ in LH surge generation. The magnitude of the LH surge is augmented after ARC neurotoxic ablation (12), which could be blocked by dynorphin infusion in the AVPV (11), whereas the magnitude of the LH surge decreased after Kiss1^ARC^ deletion using local micro-injection of antisense kisspeptin cDNA (13). These conflicting results may be the consequence of different experimental methods for ARC malfunction, causing an imbalance in stimulatory neuropeptides, kisspeptin and NKB, and the inhibitory neuropeptide Dyn (11–13), thus resulting in opposite LH level modifications.

Our recent research showed that Kiss1^ARC^ may have a novel amplificatory role in LH surge production, and this effect is influenced by gonadal steroid milieu (36). The present study used terminal optical stimulation of Kiss1^ARC→AVPV^ to further explore the role of Kiss1^ARC^ in the LH surge. Testing different stimulation frequencies in the AVPV revealed that 20 Hz could induce the LH surge-like secretion that was not evident with 5 or 10 Hz in proestrous mice. High and sustained estrogen levels are a prerequisite for the pre-ovulatory LH surge and significant upregulation of LH-surge-related genes occurs at proestrus (37), which may result in greater sensitivity of neuronal projections to optical stimulation. As in previous studies, the timing of stimulation started at 15:30 h, during which the levels of steroids were preconditioned for the pre-ovulatory LH surge, while avoiding possible confounding spontaneous LH surges (16, 24). Our results demonstrate that 20 Hz is an effective level of stimulation of Kiss1^ARC→AVPV^, which is consistent with results from other *in vivo* neuroendocrinology studies; for example, 20 Hz stimulation of Kiss1^ARC→POA^ can generate a hot flush (19) and 20 Hz terminal stimulation of GABA^ARN→POA^ lead to a significant LH release (38).

The spontaneous LH surge on proestrus is typically expected to begin 1-1.5 h before lights off and reach a peak right around the time of lights off. This was determined by LH profiles (39) and *c-fos* expression in Kiss1^AVPV^ neurons (35). Interestingly, we did not observe spontaneous LH surge in any of the optogenetically stimulated Kiss1-cre proestrous mice during our observation period (15:00 – 20:00 h). The LH surge-like secretion induced by terminal stimulation of Kiss1^ARC→AVPV^ could affect or postpone a later spontaneous surge in the proestrous mice by potentially interferes with the circadian timing mechanism, but this remains to be investigated. However, previous study showed that the induction of a surge-like release of LH following sustained 5 Hz optic stimulation for 2 h from 14:30 to 16:30 h of Kiss1^ARC^ neuron cell bodies per se in the early afternoon of proestrus did not prevent the spontaneous LH surge. These contradictory results may be due to the impact of stimulation of different parts of the of Kiss1^ARC^ neurons. As amino acid and neuropeptide neurotransmitter release is frequency-dependent, and synaptic release is normally depressed or fatigued following repeated neuronal stimulation, our stimulation may lead to fatigue of the neurotransmitter releasable pool in Kiss1^ARC^ terminals and consumption of downstream stimulatory neurotransmitters before the spontaneous LH surge (16). Additionally, the lack of spontaneous LH surges in animals receiving optical stimulation of the Kiss1^ARC→AVPV^ terminals in the presence of the combined ionotropic glutamatergic antagonists concurs with the explanation of fatigue of terminal glutamate neurotransmitter consequent to the imposed hour long optical stimulation and this appears to be evident irrespective of the stimulation frequency as 5, 10 and 20 Hz resulted in the same phenomena.

The induced LH surge-like secretion lasted for approximately 40 min in the present study, with similar LH dynamics from peak level to return to normal basal levels as observed with Kiss1^AVPV^ (16) and GnRH^POA^ optic stimulation (40), and exogenous GnRH administration (41). However, the rising phase of the LH dynamics after Kiss1^ARC→AVPV^ terminal stimulation in the present study differ from those with Kiss1^AVPV^ cell bodies stimulation (16). The LH increased gradually with Kiss1^AVPV^ optic stimulation reaching peak levels at 30 min coincident with the duration of stimulation, followed by the decline to baseline. Whereas in the present study, the LH level peaked at 10 min after the onset of 20 Hz stimulation, followed by a decline to baseline in the presence of sustained optic stimulation. High-frequency photostimulation of neuron cell bodies reportedly induces the release of neuropeptides, in contrast to low-frequency stimulation which is thought to release neurotransmitters (42). However, terminal photostimulation differs from soma stimulation and may involve a two-step process, namely initially evoking fast neurotransmitter release, which then contributes to a neuropeptide-mediate response (43, 44). The difference in LH dynamics between two types of optical stimulation may be explained as follows. The first possible reason is fatigue of terminal neurotransmitters that could not be synthesised in such a short time, hence the declining LH levels in the presence of continued optical stimulation. Secondly, it is possible that fast neurotransmitter release could induce subsequent neuropeptide release. Kiss1^ARC^ co-express the stimulatory neuropeptides, kisspeptin and neurokinin B, and the inhibitory neuropeptide dynorphin. Although, the postulated target neurons, i.e., Kiss1^AVPV^ lack the relevant kisspeptin and NKB receptors (45, 46), dynorphin may be subsequent released following Kiss1^ARC→AVPV^ terminal stimulation and contribute to the decrease in LH levels (23). The above-mentioned potential neuropeptides may underlie the different LH dynamics.

There were a few of Kiss1^AVPV^ cell bodies (<5%) also expressing EYFP most likely due to the anterograde trafficking of the AAV9 particle down the axon, the release of the AAV9, infection of these cells in AVPV and finally Flip/excision of the construct by their Cre, to cause expression of the EYFP in the AVPV Cre+ cells (47). However, we do not think the LH surge was induced by stimulating these potentially anterograde infected Kiss1^AVPV^ cell bodies. Firstly, from **Figure 2B3**, most EYFP objects represent axons and fibers that show close apposition with Kiss1^AVPV^ neurons without co-localization. Secondly, the Kiss1^AVPV^ neurons infected with EYFP virus occupy only a small proportion (<5%). It is unlikely that this number of Kiss1^AVPV^ neurons with ChR2-EYFP could evoke an LH surge, because it was previously shown that mice failed to exhibit an LH surge when 14% of Kiss1^AVPV^ neurons were activated (48). At the same time, the LH dynamics are also different with Kiss1^AVPV^ cell bodies stimulation (16). The LH release profile, namely rapid incline and sudden drop of LH, is more suited to terminal stimulation. More precise and selective neuron subpopulation simulation combined with *in vivo* experiment may be needed to resolve this problem in the future. Moreover, the inhibition of Kiss1^ARC→AVPV^ terminals during the spontaneous LH surge may also be a great supplement to our study.

In conclusion, combining optogenetic stimulation with local neuropharmacological antagonism using optofluid devices provided direct evidence supporting functional link between Kiss1^ARC→AVPV^ and surge-like LH secretion mediated *via* glutamatergic signalling in freely behaving mice. This suggests that Kiss1^ARC^ projection to the AVPV may release glutamate as a neurotransmitter that is involved in generation of the pre-ovulatory LH surge.

## Data availability statement

The raw data supporting the conclusions of this article will be made available by the authors, without undue reservation.

## Ethics statement

The studies involving human participants were reviewed and approved by Home Office License (UK). Written informed consent to participate in this study was provided by the participants’ legal guardian/next of kin. The animal study was reviewed and approved by Home Office License (UK). Written informed consent was obtained from the owners for the participation of their animals in this study.

## Author contributions

XS, YL, XL, QL, and YK designed research. XS and YL performed research. XS, YL, and XL analyzed the data. YL, XS, HL, and LW prepared figures. XS, YL, XL, and KO’B wrote the paper. All the authors reviewed the manuscript. All authors read and approved the final manuscript.

## Funding

Project of National Natural Science Foundation of China (Project Number: 82001502 to YL, 82071603 to LW), UKRI: BBSRC (BB/S000550/1), and Grants from Shanghai First Maternity and Infant Hospital (Project Number: 2021A20).

## Acknowledgments

We gratefully acknowledge all the laboratory staffs and BSU staffs employed in King’s College London.

## Conflict of interest

The authors declare that the research was conducted in the absence of any commercial or financial relationships that could be construed as a potential conflict of interest.

## Publisher’s note

All claims expressed in this article are solely those of the authors and do not necessarily represent those of their affiliated organizations, or those of the publisher, the editors and the reviewers. Any product that may be evaluated in this article, or claim that may be made by its manufacturer, is not guaranteed or endorsed by the publisher.
